# Functional outcomes of patients with short-segment Hirschsprung disease after transanal endorectal pull-through

**DOI:** 10.1186/s12876-021-01668-x

**Published:** 2021-02-23

**Authors:** Gunadi, Gabriele Ivana, Desyifa Annisa Mursalin, Ririd Tri Pitaka, Muhammad Wildan Zain, Dyah Ayu Puspitarani, Dwiki Afandy, Susan Simanjaya, Andi Dwihantoro, Akhmad Makhmudi

**Affiliations:** Pediatric Surgery Division, Department of Surgery, Faculty of Medicine, Public Health and Nursing, Universitas Gadjah Mada/Dr. Sardjito Hospital, Jl. Kesehatan No. 1, Yogyakarta, 55281 Indonesia

**Keywords:** Age at pull-through performed, Hirschsprung disease, Post-operative complications, Transanal endorectal pull-through

## Abstract

**Background:**

Transanal endorectal pull-through (TEPT) is considered the most preferable treatment method for Hirschsprung disease (HSCR) since it is less invasive and has fewer morbidities than transabdominal pull-through. Here, functional outcomes in short-segment HSCR patients after TEPT were assessed and associated with the prognostic factors.

**Methods:**

Krickenbeck classification was used to assess the functional outcomes in patients with HSCR after TEPT surgery at our institution from 2012 to 2020.

**Results:**

Fifty patients were involved in this study. Voluntary bowel movement (VBM) was achieved in 82% of subjects. Nine (18%) subjects had soiling grade 1, while two (4%) and two (4%) patients suffered constipation that was manageable with diet and laxative agents, respectively. Patients who underwent TEPT at ≥ 4 years old tended to have soiling more than patients who underwent TEPT at < 4 years old (OR = 16.47 [95% CI 0.9–301.61]; *p* = 0.06), whereas patients with post-operative complications had 10.5-fold higher risk for constipation than patients without post-operative complications (*p* = 0.037; 95% CI 1.15–95.92). Multivariate analysis showed male sex was significantly associated with VBM (OR = 9.25 [95% CI 1.34–63.77]; *p* = 0.024), while post-operative complications were strongly correlated with constipation (OR = 10 [95% CI 1.09–91.44]; *p* = 0.04).

**Conclusions:**

The functional outcomes of HSCR patients after TEPT in our institution are considered relatively good. Moreover, the VBM, soiling, and constipation risk after TEPT might be affected by sex, age at TEPT performed, and post-operative complications, respectively, while the age at TEPT performed might not be associated with functional outcomes. Further multicenter studies with a larger sample size are necessary to clarify and confirm our findings.

## Introduction

Hirschsprung disease (HSCR) is a complex genetic anomaly, characterized by the absence of ganglion cells at the myenteric and submucosal plexus of the intestines, resulting in functional obstruction [1]. HSCR can be classified based on aganglionosis length into three categories: (1) short-segment, (2) long-segment, and (3) total colon aganglionosis [1, 2]. The incidence of HSCR is about 1:5000 per live births [1, 4], while in Yogyakarta, Indonesia, its incidence is 1:3,250 live births [3].

The goal of surgical management for HSCR is to remove the aganglionic colon and make an anastomosis above the dentate line to re-establish bowel continuity [5]. Transanal endorectal pull-through (TEPT) is considered the most preferable treatment method for patients with HSCR since it is less invasive and has fewer complications than transabdominal pull-through [5–8].

Normal voluntary bowel movement (VBM), with absence of soiling and constipation are good markers of functional outcomes after surgical management of patients with HSCR [2]. Several prognostic factors have been associated with the functional outcomes after TEPT and showed inconclusive findings, including sex and age at TEPT performed [9–11]. The aim of this study was to evaluate the functional outcomes of patients with HSCR after TEPT procedure and associate them with prognostic factors, such as sex, nutritional status, age at TEPT performed, and post-operative complications.

## Methods

### Patients

This retrospective study was conducted on children < 18 year of age with HSCR who underwent TEPT procedure from January 2012 to June 2020. Patients with HSCR were diagnosed according to clinical manifestations, contrast enema, and histopathological findings [2, 3]. We included all patients with HSCR below 18 years old who underwent TEPT at our institution, while the exclusion criteria were: patients with syndromic HSCR, incomplete medical records, no histopathological findings, and TEPT performed outside of our institution.

This study was approved by the Medical and Health Research Ethics Committee of the Faculty of Medicine, Public Health and Nursing, Universitas Gadjah Mada/Dr. Sardjito Hospital, Yogyakarta, Indonesia (#KE/FK/0880/EC/2018) and written informed consent was obtained from the patients’ parents.

### Transanal endorectal pull-through (TEPT)

The patients underwent TEPT after at least three days of bowel preparation, including rectal irrigation. The TEPT was performed by three pediatric surgeons with various experiences in our institution, i.e. the number of cases performed by each pediatric surgeon were 69, 45, and 21 patients, respectively. The procedure was conducted according to previous studies [5, 12]. Everting sutures were performed throughout the anus to reveal the anal mucosa. The mucosa, then, was incised circumferentially approximately 0.5 cm above the dentate line by a needle tip cautery, followed by a submucosal dissection proximally for approximately 1–2 cm and converted to full thickness of rectal wall until the transition zone was identified. Once the ganglion cells were confirmed present in the colon on a frozen section, at least an additional 5 cm of colon was removed to be sure that the transition zone was resected together with the aganglionic segment, followed by a colo-anal anastomosis.

### Functional outcomes

The surveys of functional outcomes were conducted at follow-up visits with the median length of follow-up of 30.7 (IQR, 66.4–80.3) months. This study used the Krickenbeck classification to evaluate the functional outcomes, consisting of VBM, soiling and constipation, in patients with HSCR after TEPT who were at least three years old [2, 13, 14]. VBM is defined as feeling of urge, capacity to verbalize, and withhold the bowel movement; soiling consists of grade 1 (occasionally [once or twice per week]), grade 2 (every day, no social problem), and grade 3 (constant, social problem); whereas constipation is determined as grade 1 if manageable with diet, grade 2 if requires laxatives, and grade 3 if resistant to treatment with diet and laxatives [2, 13, 14].

### Prognostic factors

We associated the functional outcomes of HSCR patients after TEPT with the following prognostic factors: sex, age at TEPT performed, nutritional status and post-operative complications.

We divided the age at TEPT performed into two categories: < 4 and ≥ 4 years old according to a previous report [15]. We determined the nutritional status of children < 5 and ≥ 5 years old using weight-for-age z scores (WAZ) and body mass index (BMI)-for-age in relation to growth standards of the age and sex based on the World Health Organization (WHO) growth chart, respectively. Moreover, the following post-operative complications were noted, including surgical site infection, rectal mucosal prolapse and enterocolitis. The diagnosis of enterocolitis was established using the Hirschsprung-associated enterocolitis (HAEC) scoring system with the cut-off value of ≥ 10 [16].

### Statistical analysis

We presented data as frequency (percentage) and median (interquartile range, IQR). Since the number of each post-operative complications very few, they were classified as either in the presence or absence group for their association with the functional outcomes after pull-through. The association between variables was analyzed using Fisher-exact or chi-squared tests with 95% confidence interval (CI), followed by multivariate logistic regression analysis. The *p*-values < 0.05 were considered significant. The statistical analysis was performed using IBM Statistical Package for the Social Sciences (SPSS) version 21 (Chicago, USA).

## Results

### Baseline characteristics

We examined 55 medical records of patients who were recruited consecutively and excluded 5 subjects due to incomplete medical records, i.e. no information on functional outcomes. In total, we checked 50 subjects in the final analysis. Most of the subjects (68%) were males, with male to female ratio of 2.2:1. Most patients (62%) underwent TEPT at ≥ 4 years old (Table 1).

**Table 1:** Clinical characteristics of patients with HSCR who underwent TEPT in our institution

Characteristics	N (%)
Sex	
Male	34 (68)
Female	16 (32)
Nutritional status	
Well-nourished	31 (62)
Under-nourished	17 (34)
Unknown	2 (4)
Age at TEPT performed	
< 4 years old	19 (38)
≥ 4 years old	31 (62)
Post-operative complications	
Absence	44 (88)
Presence	
Enterocolitis	4 (8)
Rectal mucosal prolapse	1 (2)
Surgical site infection	1 (2)

HSCR, Hirschsprung disease; TEPT, transanal endorectal pull-through

### Functional Outcomes

VBM was achieved in 82% of subjects. Nine (18%) subjects had soiling grade 1, while two (4%) and two (4%) patients suffered constipation that was manageable with diet and laxative agents, respectively (Table 2).

**Table 2:** Functional outcomes of patients with HSCR after TEPT

Functional outcomes	N (%)
VBM	41 (82)
Soiling	
Grade 1	9 (18)
Grade 2	0
Grade 3	0
Constipation	
Grade 1	2 (4)
Grade 2	2 (4)
Grade 3	0

HSCR, Hirschsprung disease; TEPT, transanal endorectal pull-through; VBM, voluntary bowel movement

### Association between functional outcomes and prognostic factors

Subsequently, we analyzed the association between prognostic factors, including sex, nutritional status, age at TEPT performed and postoperative complication with VBM, soiling, and constipation. No associations were noted between sex, nutritional status, age at TEPT performed nor post-operative complications with VBM in HSCR patients after TEPT (*p* > 0.05) (Table 3).

**Table 3:** Association between VBM and prognostic factors in patients with HSCR after TEPT

Variables	VBM		OR (95% CI)	*p*-value
	Yes	No		
Sex			3.41 (0.77–15.06)	0.106
Male	30	4		
Female	11	5		
Nutritional status			0.89 (0.19–4.13)	0.885
Under-nourished	14	3		
Well-nourished	25	6		
Age at TEPT performed			6.26 (0.72–54.75)	0.097
≥ 4 years old	23	8		
< 4 years old	18	1		
Post-operative complications			2.64 (0.40–17.32)	0.311
Presence	4	2		
Absence	37	7		

CI, confidence interval; HSCR, Hirschsprung disease; OR, odds ratio; TEPT, transanal endorectal pull-through; VBM, voluntary bowel movement

Patients who underwent TEPT at ≥ 4 years old tended to have soiling more than patients who underwent TEPT at < 4 years old (OR = 16.47 [95% CI 0.9–301.61]; *p* = 0.059) (Table 4).

**Table 4:** Association between soiling and prognostic factors in patients with HSCR after TEPT

Variables	Soiling		OR (95% CI)	*p*-value
	No	Yes		
Sex			3.41 (0.77–15.06)	0.106
Female	11	5		
Male	30	4		
Nutritional status			0.46 (0.08–2.5)	0.367
Under-nourished	15	2		
Well-nourished	24	7		
Age at TEPT performed			16.47 (0.9–301.61)	0.059
≥ 4 years old	22	9		
< 4 years old	19	0		
Post-operative complications			0.9 (0.09–8.8)	0.928
Presence	5	1		
Absence	36	8		

CI, confidence interval; HSCR, Hirschsprung disease; OR, odds ratio; TEPT, transanal endorectal pull-through

Moreover, patients with post-operative complications had 10.5-fold higher risk to have constipation than patients without post-operative complications (*p* = 0.037; 95% CI 1.15–95.92) (Table 5).

**Table 5:** Association between constipation and prognostic factors in patients with HSCR after TEPT

Variables	Constipation		OR (95% CI)	*p*-value
	No	Yes		
Sex			4.87 (0.25–96.1)	0.298
Male	30	4		
Female	16	0		
Nutritional status			1.93 (0.25–15.12)	0.530
Under-nourished	15	2		
Well-nourished	29	2		
Age at TEPT performed			1.93 (0.19–20)	0.582
≥ 4 years old	28	3		
< 4 years old	18	1		
Post-operative complications			10.5 (1.15–95.92)	0.037*
Presence	4	2		
Absence	42	2		

*p < 0.05 is considered significant; CI, confidence interval; HSCR, Hirschsprung disease; OR, odds ratio; TEPT, transanal endorectal pull-through

### Multivariate analysis

Multivariate analysis showed that sex was significantly associated with VBM with OR of 9.25 (95% CI 1.34–63.77; *p* = 0.024), while post-operative complications were strongly correlated with constipation with OR of 10 (95% CI 1.09–91.44; *p* = 0.04) (Table 6).

**Table 6:** Multivariate analysis of the association between prognostic factors and functional outcomes in patients with HSCR after TEPT

Variables	VBM		Soiling		Constipation	
	OR (95% CI)	*p*	OR (95% CI)	*p*	OR (95% CI)	*p*
Sex	9.25 (1.34–63.77)	0.024*	0.24 (0.05–1.09)	0.064	-	0.998
Nutritional status	1.47 (0.26–8.29)	0.663	0.23 (0.03–1.69)	0.148	2.33 (0.22–25.15)	0.484
Age at TEPT performed	8.26 (0.8–85.52)	0.076	-	0.998	1.11 (0.08–16.33)	0.940
Post-operative complications	5.09 (0.52–50.13)	0.163	1.07 (0.07–14.44)	0.959	10 (1.09–91.44)	0.041*

*p < 0.05 is considered significant; CI, confidence interval; HSCR, Hirschsprung disease; OR, odds ratio; TEPT, transanal endorectal pull-through; VBM, voluntary bowel movement

## Discussion

Here, we report the functional outcomes of patients with HSCR after TEPT over an 8 years’ period of study. Our study shows that the VBM in patients with HSCR after TEPT were related to their sex. Our male patients showed a higher possibility to have the VBM. Previous study revealed that male patients had a higher risk for abnormal defecation frequency than female patients, although the overall bowel function score was similar between both sexes [17]. Prato et al*.* [18] also showed that male patients tended to have worse outcomes after surgery. Interestingly, several studies supported the hypothesis that there is an impact of sex on the outcomes of pediatric patients with various disorders [17, 19, 20]. These differences might be due to the anatomical differences between males and females, such as the pelvis and pelvic floor [18]. In contrast, Bjørnland et al*.* [21] revealed that bowel function scores were similar between male and female patients after TEPT.

Our patients with post-operative complications showed a ~ tenfold higher risk to have constipation than the patients without post-operative complications. Interestingly, children with enterocolitis had better outcomes according to HAEC score than those children without enterocolitis after pull-through [22]. Age at TEPT performed was not associated with constipation (Table 5). This finding is compatible with a previous report that constipation was not affected by the age at pull-through performed [10].

Notably, our study showed patients who underwent TEPT at ≥ 4 years old tended to have soiling more than patients who underwent TEPT at < 4 years old (Table 4). A previous study showed that age at TEPT performed was not associated with the functional outcomes [9], while other reports concluded that TEPT performed at younger age had poorer functional outcomes than older age [10, 11]. Several studies revealed that TEPT is safe and feasible not only for neonates and infants, but also for older children, adolescents and adults [15, 23–25]. In addition, no universal guidelines are established for the best age for pull-through for patients with HSCR [9]. Interestingly, while a previous study showed that the overall bowel function improved with increasing age [21], a recent report revealed a contrasting finding that the rates of soiling and constipation were not improved with increasing age [26]. Further study is important and it will be interesting to confirm whether the functional outcomes in our patients will change over time or not.

The functional outcomes of our patients were not affected by their nutritional status. Previous study noted that perioperative malnutrition had an impact on functional outcomes after pull-through [27]. This difference in findings might be due to the small sample size of our study.

The VBM, soiling and constipation frequencies in our study were ~ 82%, 18% and 8%, respectively. Our findings were compatible with previous studies that showed the frequency of soiling and constipation differed among studies between 1–60% and 9–42%, respectively [13, 28]. Soiling is the most common functional problem after TEPT (54% *vs.* 18% [our study]), while constipation is only a minor problem after TEPT (9% *vs.* 8% [our study]) [8]. However, some studies revealed that soiling only occurred in a few or none of the patients with HSCR after TEPT [29–31]. These discrepancies might be due to several reasons: (a) differences in definition of soiling among studies; (b) bias of attending pediatric surgeon during the soiling assessment since they are involved in the patients’ management; and/or (c) differences in the number of study participants [21].

Some limitations were noted in our study, such as the small sample size, particularly after stratifying into the two age groups. This approach likely leads to the study becoming under-powered to recognize statistically significant differences between functional outcomes and possible prognostic factors. Moreover, our findings are determined according to overall means without considering other factors that might affect the results, including the number and the experience of the pediatric surgeons, becoming another weakness of our study. In addition, the functional outcomes were determined at the last follow-up visits of patients at the outpatient clinic. However, the number of follow-up visits were different among patients. These facts should be considered during the interpretation of our findings since the number of follow-up visits differences might affect the results of functional outcomes.

Our study focuses on the functional outcomes in short-segment HSCR patients after TEPT and associates them with the prognostic factors, including post-operative complications. A future study is important to determine the most successful intervention in terms of post-operative complications and in functional terms.

## Conclusions

The functional outcomes of patients with HSCR after TEPT in our institution are considered relatively good. Moreover, the VBM and constipation risk after TEPT might be affected by sex and post-operative complications, respectively, while the age at TEPT performed might not be associated with any functional outcomes. Further multicenter studies with a larger sample size are necessary to clarify and confirm our findings.

## Data Availability

All data generated or analyzed during this study are included in the submission. The raw data are available from the corresponding author on reasonable request.

## Abbreviations

BMI: Body mass index
CI: Confidence interval
HAEC: Hirschsprung-associated enterocolitis
HSCR: Hirschsprung disease
OR: Odds ratio
TEPT: Transanal endorectal pull-through
VBM: Voluntary bowel movement
WHO: World Health Organization
